# A novel inactivated virus system (InViS) for a fast and inexpensive assessment of viral disintegration

**DOI:** 10.1038/s41598-022-15471-5

**Published:** 2022-07-08

**Authors:** Lea A. Furer, Pietro Clement, Gordon Herwig, René M. Rossi, Farien Bhoelan, Mario Amacker, Toon Stegmann, Tina Buerki-Thurnherr, Peter Wick

**Affiliations:** 1 Empa, Swiss Federal Laboratories for Materials Science and Technology, Laboratory for Particles-Biology Interactions, 9014 St. Gallen, Switzerland; 2 Empa, Swiss Federal Laboratories for Materials Science and Technology, Laboratory for Biomimetic Membranes and Textiles, 9014 St. Gallen, Switzerland; 3 Mymetics BV, 2333 CH Leiden, The Netherlands; 4 Mymetics SA, 1066 Epalinges, Switzerland; 5 Department of Pulmonary Medicine, Bern University Hospital, University of Bern, 3012 Bern, Switzerland

**Keywords:** Virology, Characterization and analytical techniques

## Abstract

The COVID–19 pandemic has caused considerable interest worldwide in antiviral surfaces, and there has been a dramatic increase in the research and development of innovative material systems to reduce virus transmission in the past few years. The International Organization for Standardization (ISO) norms 18,184 and 21,702 are two standard methods to characterize the antiviral properties of porous and non-porous surfaces. However, during the last years of the pandemic, a need for faster and inexpensive characterization of antiviral material was identified. Therefore, a complementary method based on an Inactivated Virus System (InViS) was developed to facilitate the early-stage development of antiviral technologies and quality surveillance of the production of antiviral materials safely and efficiently. The InViS is loaded with a self-quenched fluorescent dye that produces a measurable increase in fluorescence when the viral envelope disintegrates. In the present work, the sensitivity of InViS to viral disintegration by known antiviral agents is demonstrated and its potential to characterize novel materials and surfaces is explored. Finally, the InViS is used to determine the fate of viral particles within facemasks layers, rendering it an interesting tool to support the development of antiviral surface systems for technical and medical applications.

## Introduction

What started as a mysterious and unknown lung disease in Wuhan in December 2019 has evolved into a serious pandemic similar to the "Spanish flu": COVID-19. The trigger is the beta-coronavirus SARS-CoV-2, which is responsible for various symptoms such as inflammation of the throat and respiratory tract, coughing, shortness of breath, fatigue, fever, myalgia, conjunctivitis, loss of smell and impaired taste. In severe cases, it can lead to acute lung failure, multi-organ failure and death^1^.

As of June 2022, the number of confirmed COVID-19 cases worldwide has reached 529 million and the number of deaths has risen to over 6 million^2^. Although effective vaccines and better treatment strategies have been developed in the meantime, new highly contagious mutations and low vaccination rates in poor countries continue to force the health care systems to their limits. Therefore, non-pharmaceutical interventions such as contact reduction, hygiene and facemasks remain important to keep the propagation of the virus at bay^3^.

The overall goal of these measures is to slow down disease transmission between people. COVID-19 spreads mainly by respiratory droplets among people who are in close contact with each other^4^. However, also other mechanisms of disease transmission are also possible. For example, aerosol transmission can occur indoors in crowded and poorly ventilated spaces, and it is also possible to catch COVID-19 indirectly through touching surfaces or objects contaminated with the virus from infected people, followed by touching the eyes, nose or mouth (fomite transmission)^4^. It has been shown that SARS-CoV-2 is stable from a few hours to days depending on the chemistry of the surface on which the virus is deposited. For example, van Doremalen et al*.* demonstrated that SARS-CoV-2 remained stable on plastic and stainless steel surfaces for several hours. Viable virus could be detected up to 72 h after application to these surfaces, and the half-life of SARS-CoV-2 was estimated to be 5.6 h on stainless steel and to 6.8 h on plastic^5^. In contrast, no viable virus could be detected on cardboard surfaces after 24 h, and copper surfaces tended to inactivate the virus within 4 hours^5^. Chin et al*.* confirmed that SARS-CoV-2 is more stable on smooth and hydrophobic surfaces. While no infectious virus could be recovered from printing and tissue culture papers after 3 h, viable and infectious virus was still present on treated smooth surfaces (glass and banknotes) after 2 days, on stainless steel and plastic after 4 days and on the hydrophobic outer layer of a surgical mask after 7 days^6^.

Since this high surface stability increases the risk of smear infection, it would be highly beneficial if virus particles that reach vulnerable surfaces such as touch screens, doorknobs, ventilation filters, textiles, train seats or handrails could be inactivated immediately without further disinfection processes. The same applies also to virus particles deposited on facemasks, since inappropriate mask handling is a commonly faced problem.

Therefore, the development of antiviral materials, coatings and facemasks is of tremendous importance not only to fight COVID-19, but also to prevent similar pandemic or epidemic outbreaks in the future.

There are several ways to inactivate or even destroy viruses. The most known are detergents or ethanol solutions which disintegrate the lipid and protein structure of the virus^7–10^, radiation such as ultraviolet light with wavelength between 200 and 300 nm (UVC)^11–13^, thermal treatment like autoclaving^14,15^, oxidation^16,17^ or high positive surface charge^18,19^. When new antiviral materials are developed, it is crucial to investigate and quantify their potential to trap, inactivate and/or kill the viruses. Currently, there are two ISO norms available, which regulate the measurement of antiviral activity on plastics and other non-porous surfaces (ISO 21702) and the determination of antiviral activity of textile products (ISO 18184). In both norms, the infectious virus titer needs to be determined by either a plaque assay or the Median Tissue Culture Infectious Dose (TCID_50_) method. Both of these assays require working with the real infectious virus and are based on the principle that cytopathic effects caused by the virus can be visibly assessed in vitro. Although these assays deliver reliable and comparable results on the antiviral activity of the materials, they have several disadvantages: working with real viruses such as e.g. SARS-CoV-2 requires special infrastructure (e.g. biosafety level 3 equipped labs) and trained employees. This prevents the widespread use of these analyses in most industrial and research settings and consequently delays the rapid development of novel antiviral materials as observed during the current COVID-pandemic. Consequently, novel characterisation methods are urgently needed to facilitate the fast, cheap and safe pre-screening of a large number of materials and surfaces for potential antiviral properties.

**Figure 1:**
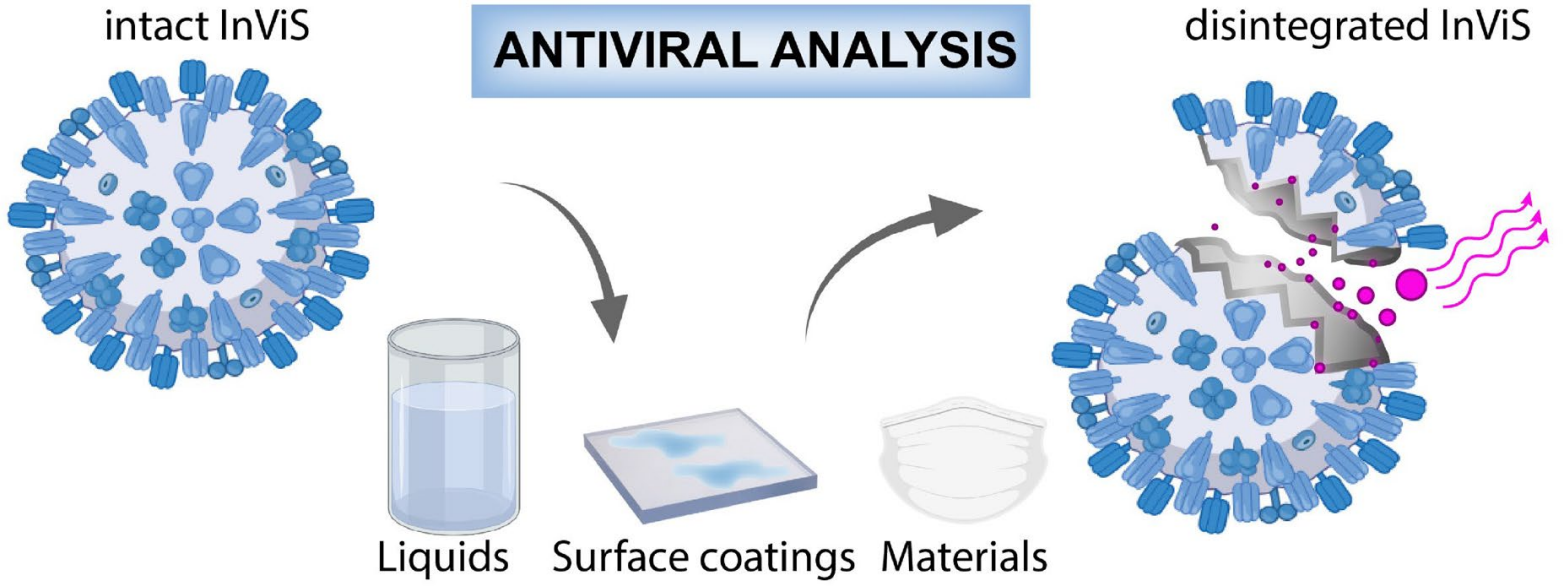
InViS, a fluorescent approach to detect viral envelope disintegration caused by antiviral materials.

In this work, an inactivated octadecylrhodamine R18 loaded A/Brisbane 59/2007 influenza virus system (InViS) is exploited as a surrogate for an infectious virus to assess viral disintegration by simple fluorescence measurements. This novel virus system allows the study of antiviral effects of different chemicals, cleaning agents and materials in a simple way: the fluorescence increases when virus particles disintegrate, since the fluorescent probe is self-quenched inside the intact virus lipid structure (Fig. 1).

In this study, we explored and defined the application areas, predictive power and limitations of this novel InViS as an alternative pre-screening method by analyzing the effect of antiviral chemicals (70% ethanol, citric acid), different potential antiviral nanoparticles (NPs) and surface coatings. In addition, we exploited the use of InViS to assess and quantify the fate and localization of virus particles in facemask layers.

## Results

### InViS characterization

The inactivation of viruses and loading with fluorescent dye^20^ are established procedures applied in vaccine research. Here, we explore the potential of such an inactivated virus in the field of antiviral characterization. The Inactivated Virus System (InViS) used in this work consists of an inactivated octadecylrhodamine (R18) labeled A/Brisbane/2007 influenza virus solution, whose fluorescence increases upon membrane rupture or fusion^21^.

**Figure 2:**
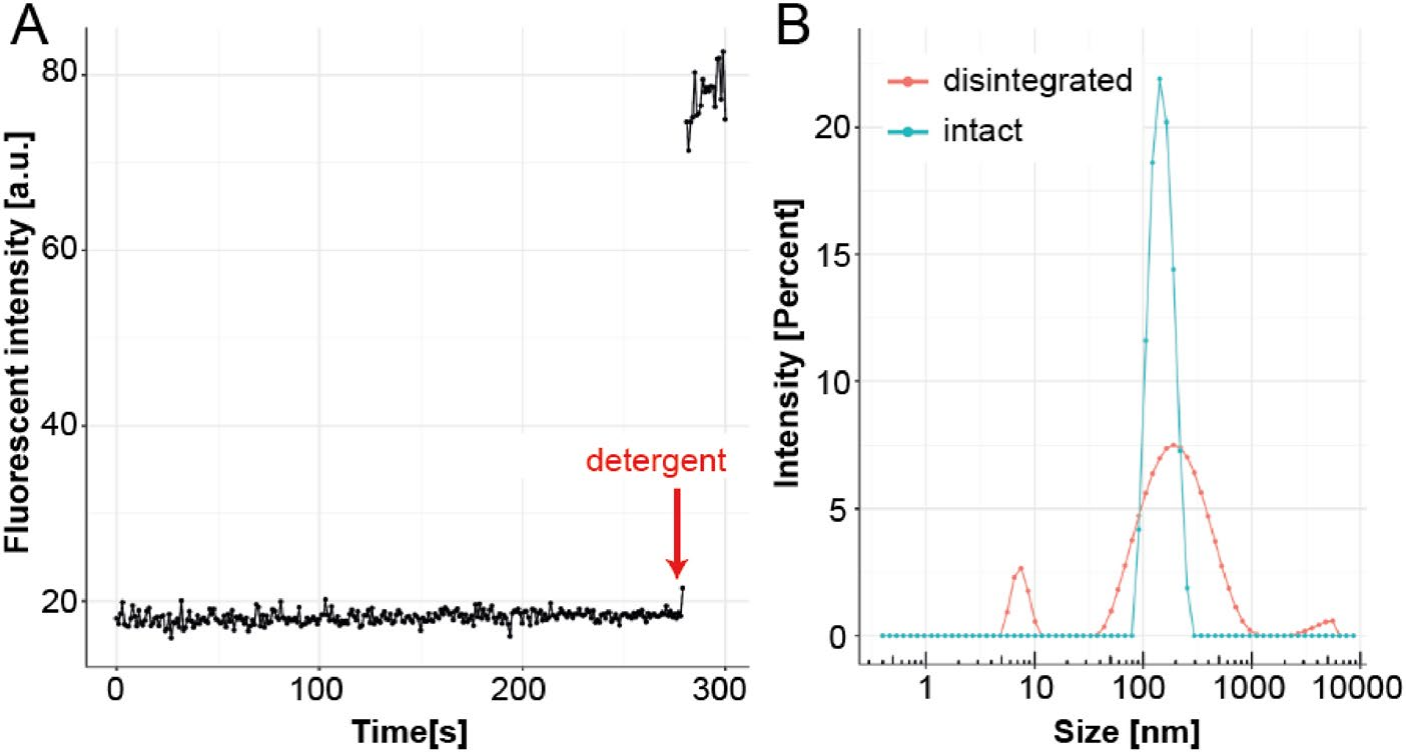
Effect of OEG on InViS. (**A**) The fluorescence intensity of InVis was monitored continuously over 300 s. After 280 s, OEG was added to the sample to induce viral disintegration and the release of the fluorescent R18 label from the viral membrane. (**B**) Particle size distribution measured by DLS before and after detergent administration. The intact InViS is highly monodisperse. When in contact with detergent, the virus disintegrates, leading to several populations of residual aggregates and a higher polydispersity index.

The measured virus concentration was 1.5 × 10^13^ (± 9.8 × 10^10^) particles mL^−1^ and the virus particles exhibited a very homogeneous size of 110.6 ± 0.8 nm. The fluorescent label inside the virus was self-quenched and the fluorescence of the intact virus was only 23% of that after addition of the detergent octaethylene glycol monododecyl ether (OEG) (Fig. 2A). Dynamic Ligth Scattering (DLS) measurements confirmed that the detergent damaged the virus particles and led to their disintegration. While the InViS system was monodisperse (polydispersity index (PdI) of 0.063 ± 0.023) before detergent administration, the addition of OEG resulted in a highly polydisperse sample (PdI of 0.443 ± 0.048) with smaller and larger peaks likely corresponding to virus fragments, and agglomerated viral structures and detergent micelles (Fig. 2B). Therefore, the InViS system could be used to assess viral disintegrating properties of chemicals and materials by a fast and simple fluorescence readout. The detection limit (defined as the particle concentration at which the viral disintegration is no longer detectable by an increase in fluorescence from the instrument baseline response) was determined by serial dilutions and was 10^8^ particles mL^−1^.

### Effect of known antiviral liquids

**Figure 3:**
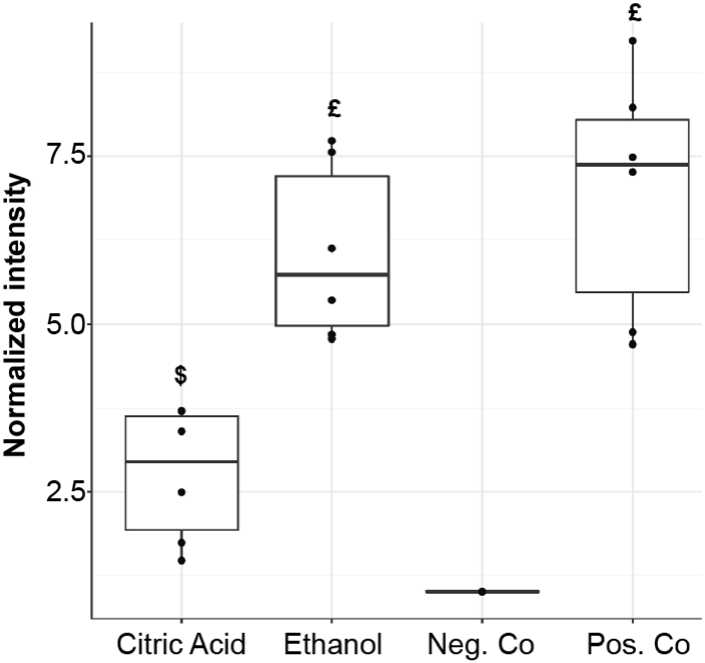
Fluorescence intensity of InViS after incubation with citric acid (1 M) and 70% ethanol. InViS in PBS and InViS treated with OEG served as negative and positive control, respectively. Results represent the mean and corresponding standard deviations from three independent experiments with two replicates each. ^$^p < 0.01, ^£^p < 0.001 compared to negative controls.

To verify that InViS is not only sensitive to detergents, but can also detect other known antiviral compounds with different antiviral potency, we performed further experiments with 70% ethanol and citric acid (1 M). Both chemicals induced a significant increase in fluorescence signal intensity (Fig. 3). However, the mild antiviral agent citric acid^22^ (pH 2.95) only induced a partial release of the R18 label, while an almost complete release similar to the positive detergent control was detected upon addition of 70% ethanol.

### Assessment of potential antiviral NPs

**Figure 4:**
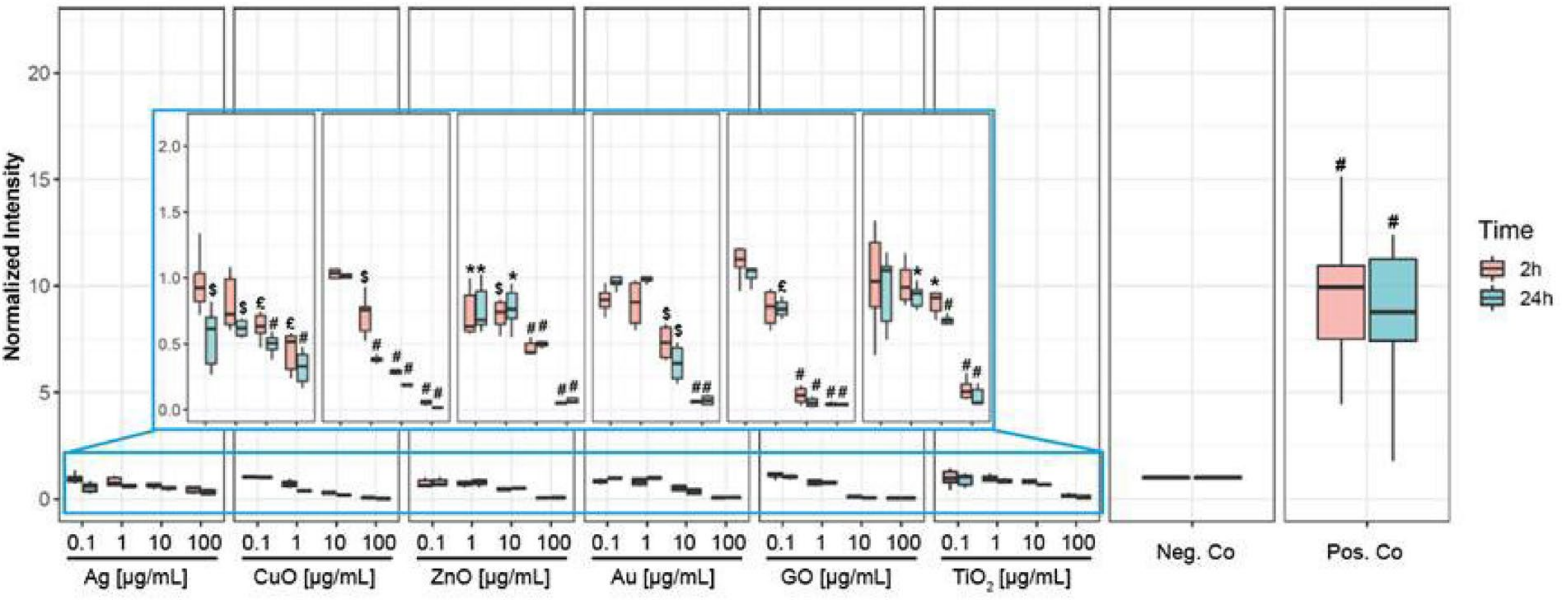
Fluorescence intensity after incubating InViS with NPs. NP suspensions were incubated for 2 and 24 h with InViS solution and suspensions were then centrifuged to remove NPs before fluorescence measurements. Negative control is InViS in PBS (0.4% v/v) and positive control is InViS in PBS (0.4% v/v) and OEG (1.25 mg mL^−1^). Results represent the mean and corresponding standard deviations from at least three independent experiments with two technical replicates per experiment. *p < 0.05, ^$^p < 0.01, ^£^p < 0.001 and ^#^p < 0.0001 compared to negative control.

Different studies have shown that several NPs including copper oxide (CuO), zinc oxide (ZnO), titanium dioxide (TiO_2)_, gold (Au), silver (Ag), selenium (Se) and graphene oxide (GO) possess antiviral properties^23–29^, yet the underlying toxicity mechanisms are often not fully understood. To investigate if the antiviral mechanisms of these NP types may include viral disintegration, we incubated the InViS with different NP concentrations for 2 and 24 h and measured the fluorescence intensities to detect the potential release of the fluorescent R18 label. None of the investigated NPs were able to destroy the InViS envelope, since fluorescence levels did not increase and were comparable to the negative control (intact InViS without NPs) (Fig. 4). At increasing NP concentrations, there was even a decrease in fluorescence intensity compared to untreated control samples. Although NPs were removed by centrifugation before the fluorescence measurements to avoid potential interference responses, we performed further interference studies, which confirmed the absence of NP autofluorescence signals or non-specific effects on the fluorescence measurements (data not shown).

### Assessment of non-porous antiviral surfaces

**Figure 5:**
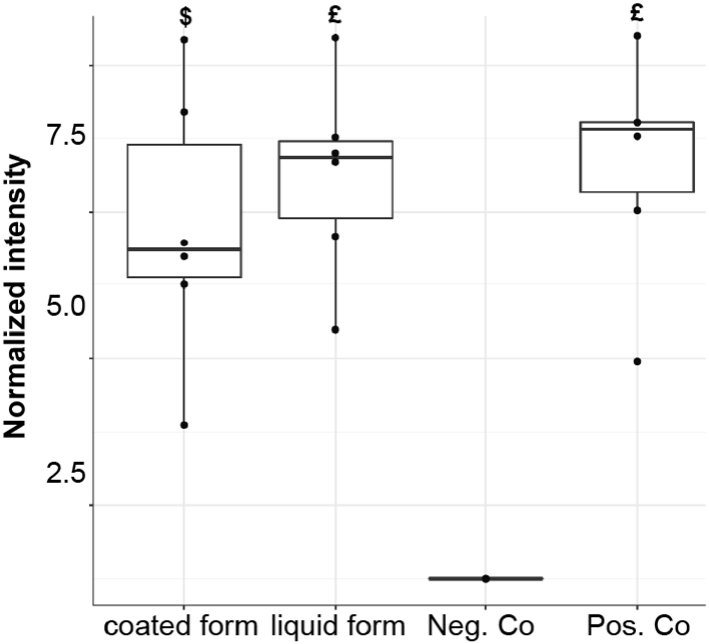
Fluorescence intensity of InViS after contact with an antiviral coating. Both the liquid and coated form of an antiviral coating solution (patent number PCT/EP2021/060580^30^) lead to a significant increase in fluorescence, indicating virus capsule disintegration. Fluorescence levels of the intact InViS in PBS and InViS incubated with OEG served as negative and positive control, respectively. Results represent the median and corresponding standard deviations from three independent experiments with two technical replicates. ^$^p < 0.01 and ^£^p < 0.001 compared to negative control.

After evaluating the InViS system for the detection of antiviral effects of chemicals and NPs, we further assessed its suitability to detect the antiviral potential of surfaces and coatings. To obtain a flat and non-porous antiviral surface, sterile cell culture plates were coated with a novel antiviral coating solution (patent number PCT/EP2021/060580^30^). The InViS was brought in contact with either the coated surface or the liquid form of the coating solution and a significant increase in fluorescence intensity could be detected in both cases (Fig. 5), clearly indicating virus capsule disintegration.

### Localization of InViS within multilayer system

Besides detecting virus disintegration, we hypothesized that the InViS system can also be suited to detect the fate and localization of virus particles in multilayered facemasks and textiles, which is highly valuable information to understand which layer(s) need to exhibit strong antiviral activity. We used a filtration efficiency test system to expose textile community masks, surgical masks and FFP2 (as defined in EN149 standard, corresponding to US-standard N95 respirator) masks to aerosols containing InViS. As a comparison, filtration efficiency testing was also performed with salt solutions, which represents the current European standard procedure (EN13274-7) to assess the filtration efficiency of textiles. After the filtration testing, the different mask layers were peeled apart and the localization of the particles was assessed by Scanning Electron Microscope (SEM) (salt and InViS; textile community masks only) and fluorescence measurements (InViS only; all mask types).

**Figure 6:**
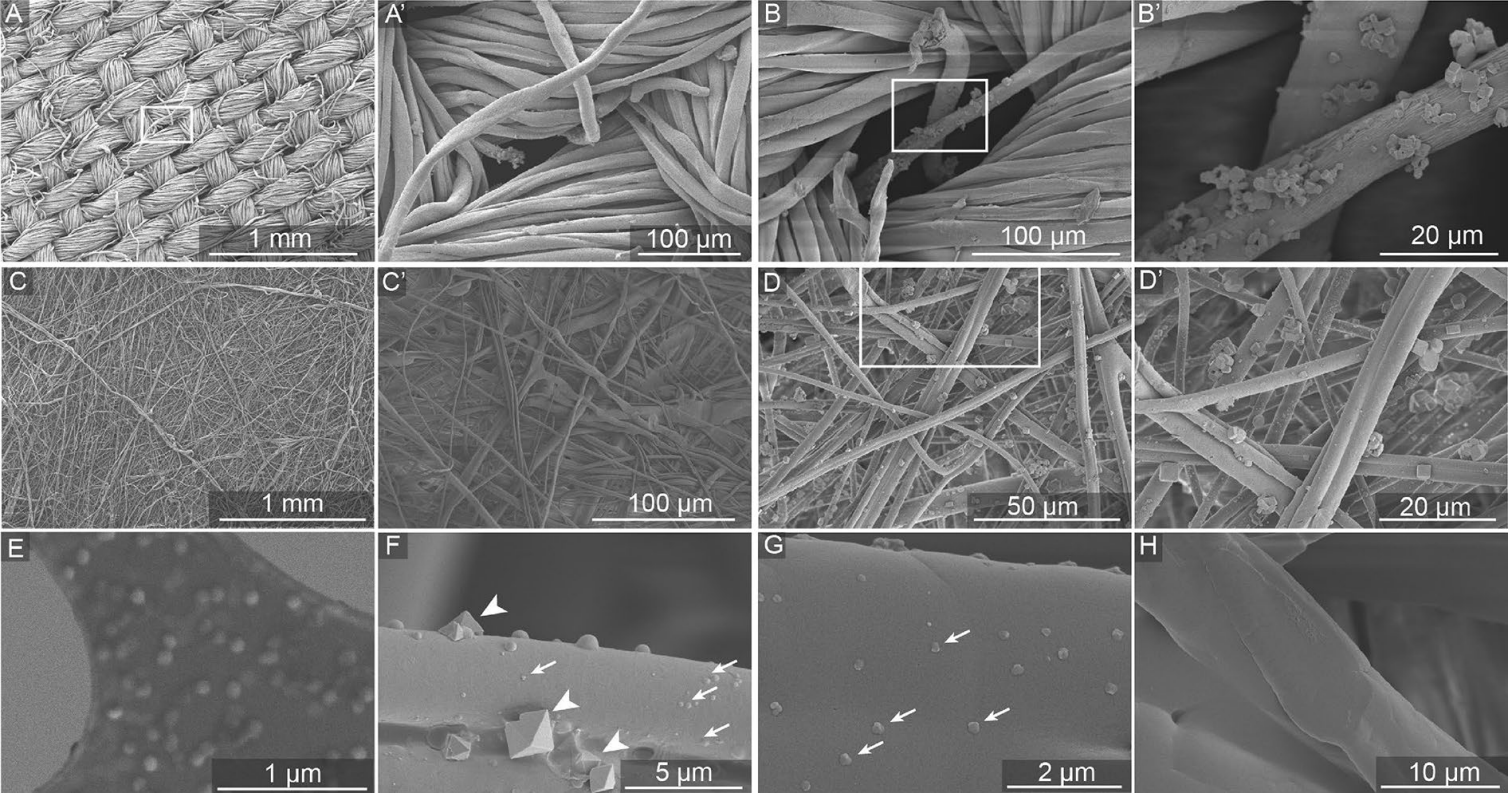
Localization of NaCl and InViS particles in textile community masks after filtration efficiency tests. Micrographs after filtration efficiency test with NaCl show: (**A**) the outermost woven layer of a textile community mask, (**B**) a pore of the outermost woven layer after the filtration test with NaCl particles, (**C**) the inner meltblown fitration layer and (**D**) salt particles filtered by the inner meltblown filtration layer. (**A'**–**D'**) represent magnifications of the white quadrant in (**A**–**D**) (except **C'** which is from another area not visible in **C**). Micrographs after filtration efficiency test with InVIS show: (**E**) SEM imaged of InViS on Transmission Electron Microscope (TEM) grid sample holder, (**F**) and (**G**) the inner meltblown filtration layer (salt crystals and InViS particles are highlighted with arrow heads and arrows, respectively). (**H**) is a reference fiber from the inner meltblown layer which did not undergo filtration experiments.

Micrographs of the outer woven layer (Fig. 6A,A',B) and the inner meltblown layer (Fig. 6C,C',D,D',F–H) of a community mask are shown before and after filtration efficiency tests with salt or InViS particles. Additionally, SEM of InViS on a TEM grid (Fig. 6E) was included, to facilitate the identification of viral particles in the mask layers. The InViS particles appeared monodisperse with a size of approximately 100 nm, while salt crystals appeared larger.

**Figure 7:**
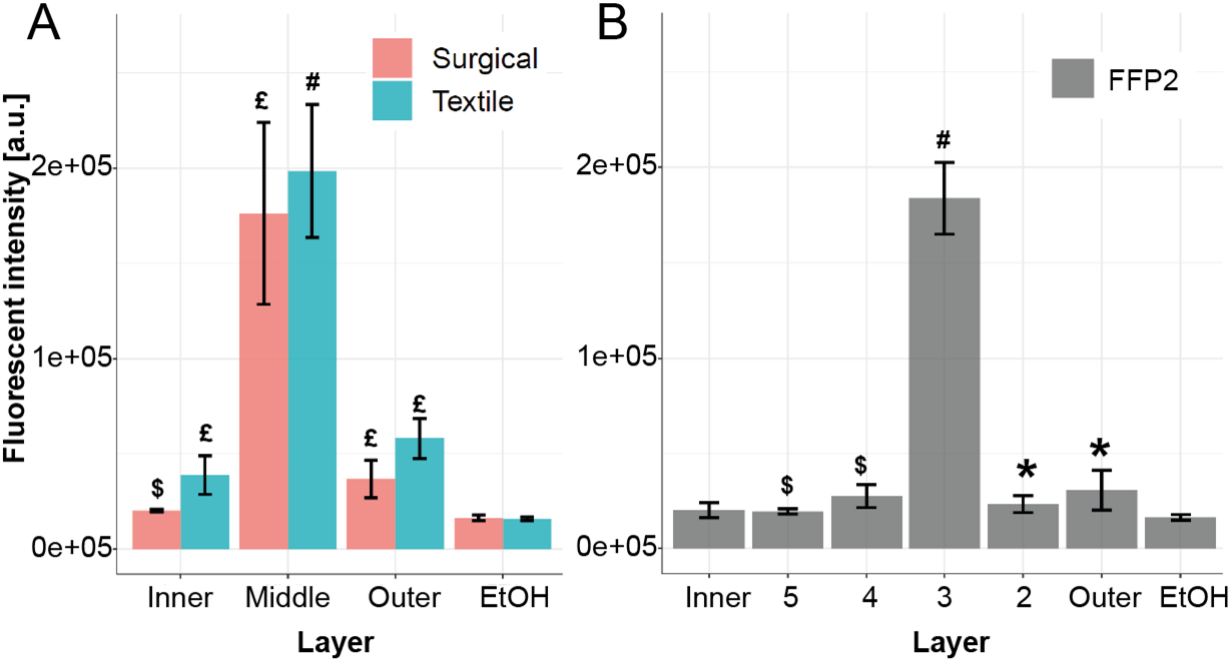
Fluorescent intensity of the different layers of a textile, surgical (**A**) and FFP2 (**B**) mask after filtration efficiency testing with InViS. The air stream flowed from right to left (from outer to inner layer). EtOH control is the fluorescence value of the ethanol solution only. Results represent the mean and corresponding standard deviations from three independent experiments with two technical replicates. *p < 0.05, ^$^p < 0.01, ^£^p < 0.001 and ^#^p < 0.0001 compared to controls.

During the filtration efficiency experiments, the air was first passed through the pores in the outer woven layer of the masks, where a preferential accumulation of salt particles near the pores was observed while most of the outer layer fibers were devoid of particles (Fig. 6B,B'). InViS particles were not observed in the outer woven layer. Next, the air reached the inner meltblown layer, which showed a considerable accumulation of salt (Fig. 6D) and InViS (Fig. 6F,G) particles. To further corroborate the preferential localization of InViS particles to the inner meltblown layer in a semi-quantitative manner, complementary fluorescence measurements were carried out. Therefore, the different mask layers were treated with ethanol to release the rhodamine from the adsorbed InViS. The fluorescence intensity corresponding to each layer for surgical, textile and an FFP2 facemask is shown in Fig. 7A,B. This wash out resulted in InViS disintegration, but still allowed the quantification of virus particles in each layer as the fluorescence intensity is proportional to the amount of filtered viral particles.

## Discussion

The severe COVID-19 pandemic has dramatically changed people's lives in the past 2.5 years. Things that used to be taken for granted, such as a regular job in the office, meeting friends, attending cultural events and travelling, were suddenly no longer possible. Lock-downs forced people all over the world to stay at home, and had a negative impact on the economy. Healthcare systems reached their capacity limits, and many people suffered or even died from a severe illness. To fight the pandemic, many researchers and clinicians worked under a high pressure to understand the disease and develop efficient vaccine and treatment options. In parallel, the textile industry intensively worked on the development of user-friendly, comfortable and effective facemasks to slow down disease transmission between people^31^.

Soon, it was recognized that the development of antiviral materials and surfaces was recognized as an efficient mean to slow down the spread of SARS-CoV-2 and prevent infections by direct contact. However, the development of antiviral materials is a time- and cost-intensive process. One of the major issues was that the antiviral properties of newly developed coatings and materials could only be assessed by conducting tests with the real virus (ISO 21702 and ISO 18184). This required trained employees and a special infrastructure (e.g. biosafety level 3 equipped labs) which were difficult to access for most material developers and considerably delayed the development of innovative antiviral materials.

Therefore, alternative test methods that are inexpensive as well as easy and safe to handle are highly valuable to facilitate the rapid pre-screening of novel antiviral material designs to advance innovation and rapidly identify promising candidates for further testing with the real virus. In this study, we present InViS, a novel alternative virus system that allows a fast, cheap and safe detection of virus disintegration activity of liquids, compounds and materials by simple fluorescence measurements. We used an inactivated Rhodamine-18 labeled A/Brisbane 59/2007 Influenza virus that has close structural similarities to SARS-CoV-2, namely constituting an enveloped RNA virus of ~ 100 nm in size with hemagglutinin on its surface. The virus has been inactivated with β-propiolactone, which preserves the antigenic virus structure, but renders the virus non-infectious due to alkylation of nucleic acid bases, suppression of genome replication, induction of genome degradation and protein and genome cross-linking^32^. The most interesting feature of InViS is that the fluorescent probe is self-quenched within the viral membrane. As a consequence, the fluorescence of InViS significantly increases when the virus particles disintegrate and release the fluorescent dye.

It is known that ethanol can disintegrate virus particles by lipid membrane dissolution and protein denaturation^33–35^. Similarly, a low pH can lead to membrane degradation^36–38^. Therefore, we exposed InViS to 70% ethanol and citric acid to test its sensitivity against known antiviral liquids. Indeed, both chemicals led to a significant increase in fluorescence. When the InViS particles were in contact with the ethanol solution, complete viral disintegration occurred within 5 min. In the case of citric acid solution, the viral disintegration remained partial, indicating that the InViS system can deliver (semi-)quantitative data on virus disintegration.

Because some NPs can have antiviral properties they are extensively explored for the development of novel antimicrobial materials and coatings^16,23,39^. Therefore, we investigated whether Ag, Au, CuO, ZnO, TiO_2_ and graphene oxide NPs are able to disintegrate InViS. Although antiviral effects have been described for the investigated NP types, none of the tested NPs induced viral envelope disintegration even upon prolonged exposure for up to 24 h. We even observed a slight decrease in fluorescence signals with increasing NP concentrations, which could be due to particle adsorption to and/or penetration of the viral capsule surface and subsequent removal from the liquid sample during the centrifugation step. This would be in line with results published by Kim et al., where a co-precipitation of Au NPs and Influenza A virus during centrifugation was reported^24^.

These results for the NPs are consistent with the existing literature, which suggests that antiviral activities of most NPs appear to rely on other effects than capsule disintegration. For example, Ag NPs were shown to bind or denature the viral capsid protein or inhibit the virus from binding to cell receptors, therefore preventing virus entry into the host cells^23^. NPs containing Cu could catalyze the generation of radicals via Fenton or Fenton-like reactions, oxidizing the capsid proteins and consequently blocking the viral infection at an early stage^23^. AuNPs have been shown to oxidize the disulfide bonds of the hemagglutinin glycoprotein on the viral surface, causing its inactivation and thus impeding the membrane fusion of the virus with host cells^23^. TiO_2_ NPs may damage lipids and glycoproteins in the viral envelope^40^. Graphene nanosheets were able to interrupt hydrophobic protein–protein interactions and graphene oxide could adsorb virus particles, therefore preventing their interaction with the cell membrane.

With this knowledge, the biggest limitation of our InViS system becomes evident: membrane dissolution is not the only inactivation method and therefore not all antiviral materials can be characterized using InViS. Nevertheless, many antiviral materials rely on viral envelope disintegration. The aim of the InViS is not to replace the existing and approved ISO tests. Rather, it was designed to offer a simple, cheap and safe alternative to assess viral disintegration (which prevents potential resistance development) at an early stage of research and development. The system is particularly interesting for researchers and manufacturers who want to assess the efficacy of materials that are designed to disintegrate the lipid envelope. During this study, we evaluated such an antiviral coating solution (patent number PCT/EP2021/060580^30^) with InViS. We report a strong increase in fluorescence, and confirm that the coating solution resulted indeed in viral disintegration. Such rapid tests can be highly beneficial in the development of antiviral liquids and coatings. For example, they allow the screening of a large number of different substances, compositions and concentrations to identify the most promising solution for further development.

Additionally, the InViS can be used to detect the fate and localization of NPs in multilayered structures, textiles and facemasks. We introduced InViS in a filtration efficiency system, where the performance of different types of facemasks was evaluated. This allowed the use of aerosols with biologically relevant virus particles instead of substances prescribed by the standards such as salt or oil. Due to the mixed presence of salt crystals and viral particles, it was not possible to measure the filtration efficiency directly from the virus aerosol using the particle analyzer. Nevertheless, we obtained important information regarding the accumulation of virus particles in the different mask layers and the relevance of using NaCl particles as a model for viral particles. Imaging by SEM of the different layers revealed that both salt and virus particles preferentially accumulated in the inner meltblown layer, suggesting that NaCl aerosols could be representative of virus particles despite their different characteristic in terms of size and shape. Nevertheless, an adaptation of the filtration efficiency bench could be implemented to conduct filtration efficiency tests with InViS. It would be interesting to quantify the filtration efficiency by collecting the residual viral particles that passed through the tested facemasks and filters, for example with a bubbler which allows remaining viral particles to be collected in a liquid solution. By comparing the residual fluorescence of mask samples with blank samples, variations in filtration efficiency may be detected. This would allow an evaluation of the filtration efficiency of face masks using virus particles and bring us one step closer to determining the filtration properties of medical and technical filtration systems against real viruses in a more relevant exposure scenario.

In conclusion, we report the development of a novel method assessing potential antiviral compounds and surfaces with an inactivated virus system (InViS) for a fast, inexpensive and safe assessment of virus disintegration by simple fluorescence measurements. InViS can be further used to study the fate and localization of viral particles on non-porous as well as porous materials such as technical and medical textiles, rendering it a valuable tool to support the development of novel antiviral materials, coatings and facemasks.

## Materials and methods

### Virus inactivation and fluorescent dye loading

Chemically inactivated and purified monovalent influenza virus A/Brisbane/59/2007 (H1N1) solution at GMP grade was obtained from Seqirus (Melbourne, Australia) hemagglutinin (HA) (1.6 mg mL^−1^) determined by Single Radial Immunodiffusion Assay, SRID. Rhodamine B octadecyl ester perchlorate (R18) was purchased from Merck KGaA (The Netherlands) and dissolved in HLPC grade anhydrous ethanol at a final concentration of 10 mM. R18 solution (40 µL) was added to an influenza solution (1 mL) dropwise at room temperature (RT) under continuous stirring at 200 rpm for 15 min. Non-incorporated R18 was removed by separation on a Sephadex G50 column (Merck KGaA). The void volume fraction was collected and further characterized.

### Virus characterization

Particle size, size-distribution and concentration were analyzed by Nanoparticle Tracking Analysis on a Malvern NanoSight LM10 instrument. The sample was diluted 1:10,000 in HNE buffer (HEPES (10 mM) pH 7.4, NaCl (142.5 mM), EDTA (5 mM), filtered through a 0.1 µm syringe filter before use) and injected into the analysis chamber of the 405 nm laser module with a constant flow of 70 units at a controlled temperature of 25 °C and a viscosity setting of 0.975–0976 Cp. 5 captures were performed, with each capture having a duration of 60 s. Camera setting was level 15 with a detection threshold of 3. R18 fluorescence and fusion activity were determined to confirm fluorescence quenching and the presence of HA on the virus surface as described^20,41^. For the fusion of influenza virosomes with erythrocyte ghosts, the medium was acidified to pH 4.5. R18 fluorescence was measured continuously at excitation and emission wavelengths of 560 and 590 nm, respectively. For calibration of the fluorescence scale in fusion experiments, the initial fluorescence of the labeled membranes was set to zero and the fluorescence at infinite probe dilution at 100%. The latter value was obtained after addition of OEG (Merck KGaA, The Netherlands, final concentration 1 mM). To confirm that the virus remained intact after shipment, DLS measurements were performed to determine the hydrodynamic diameter of the virus particles and their polydispersity index in PBS before and after the addition of the OEG (Zetasizer Nanoseries, Nano-ZS90, Malvern, Worcestershire, UK, 1.25 mg mL^−1^). Furthermore, fluorescence measurements of the serially diluted virus with and without OEG (1.25 mg mL^−1^, 5 min of incubation time) were performed with a Horiba FluoroMax SpectraFluorometer to establish the detection limit.

### Effects of known antiviral compounds

70% ethanol (CAS 64-17-5) and citric acid (1 M; CAS: 77-92-9) were incubated with InVis (0.4% v/v). The samples were homogenized with a vortex mixer and incubated for 5 min at RT. Fluorescence measurements were performed using a Horiba FluoroMax SpectraFluorometer. The excitation wavelength was 560 nm and emission spectra were measured between 580 and 650 nm. An InViS solution in PBS (0.4% v/v) was used as a negative control to provide the fluorescence intensity of the intact virus. As positive control, OEG (CAS: 3055-98-9; 2.5% v/v) was added to disintegrate the virus and indicate the corresponding fluorescence intensity.

### NP characterization and dispersion

To study potential antiviral effects of NPs, we used particles that were used and fully characterized in previous studies. The most relevant properties of the particles are summarized in Table 1.

**Table 1:** Characteristics of the investigated NPs.

	Primary particle size (nm)	Hydrodynamic diameter (nm)/ PdI	Zeta potential (mV)	Refs
CuO	20 (TEM)	1165/0.335 (UPW)	n.d	^42^
ZnO	15.5 ± 3.9 (TEM)	161.8/0.1 (UPW)	− 24.3 (UPW)	^43^
Ag-COONa	5–15 (TEM)	~ 110 (UPW)	− 32.55 (UPW)	^44^
Au-COONa	4.5 ± 1.5 (TEM)	n.m	− 28.8 (UPW)	^45^
GO	1–50 µm (SEM)	n.m	− 45.8 (10% PBS)	^46^
TiO_2_	5–6 (TEM)	89 ± 54.1 (PBS)	− 21.1 ± 1.2 (PBS)	^47^

Particles available as suspensions (Ag-COONa, Au-5-COONa) were diluted with ultrapure water to a stock suspension of 1 mg mL^−1^ and homogenized using a vortex mixer (1 min). Particles available as powder (CuO, graphene oxide, TiO_2_ and ZnO) were suspended in ultrapure water to a stock suspension of 1 mg mL^−1^ using a probe sonicator operating at 230 V/50 Hz (Branson Sonifier 250, Branson Ultrasonic Co., Danbury, CT, USA, probe diameter of 6.5 mm, maximum peak-to-peak amplitude of 247 μm) for 5 min at 13 W.

### Assessing antiviral effects of NPs

Different particle concentrations (0.1, 1, 10 and 100 µg mL^−1^) were incubated with a solution of InViS in PBS (0.4% v/v; stock concentration of InVis: 1.5 * 10^13^ particles mL^−1^) for 2 and 24 h. The incubation was carried out at RT in the dark and with continuous shaking. The suspensions were centrifuged for 10 min at 4500×*g* to remove the NPs. Fluorescence of the supernatants was measured with a Horiba FluoroMax SpectraFluorometer. To quantify the antiviral effect, fluorescence was compared to the fluorescence of a virus suspension in PBS (negative control) and a virus suspension treated with OEG (1.25 mg mL^−1^; positive control where all the Rhodamine should be released). To exclude autofluorescence or fluorescence quenching of the NPs, fluorescence of pure NP suspensions (without virus) and fluorescence of NPs incubated with virus and detergent were also measured.

### Effects of antiviral coating on flat, non-porous surfaces

An antiviral solution [patent number: PCT/EP2021/060580^30^] was used to create an antiviral coating. First, the antiviral properties of the liquid form were characterized with a 5 min incubation with an InViS solution (0.4% v/v) and fluorescence measurements. To characterize the properties of the coated form, a volume of 0.5 mL was spread evenly in disposable petri dishes and incubated at RT for 24 h. The ISO norm 21,702, which covers the antiviral characterization of non-porous materials, was used as a guideline for sample preparation. The virus inoculum, 0.4 mL for each sample, was composed of a 20% v/v InViS stock solution in PBS. An inoculum with InViS (20% v/v) in PBS and an inoculum with InViS in PBS (20% v/v) and OEG (62.5 mg mL^−1^) in empty petri dishes were used as a negative and positive control, respectively. An inert polymer (low density polyethylene (LDPE)) film (40 mm × 40 mm) was used to cover the inoculum. The film was gently pressed down to form a sandwich structure and maximize the contact surface area between the inoculum and the antiviral sample, while preventing leakage beyond the edges of the inert film. The samples were stored for 24 h in the dark at RT. 20 mL of PBS were added to the petri dish and the dishes underwent agitation to ensure the homogenization of the freshly added PBS and the remaining inoculum. After mixing, 2 mL of the washout were collected and pipetted into transparent cuvettes. The emission fluorescence spectra of the washout was characterized using the Horiba FluoroMax SpectraFluorometer. The excitation wavelength was set to 560 nm and the emission intensity was measured between 580 and 650 nm. For each sample, two replicates were analyzed.

### Filtration efficiency bench

**Figure 8:**
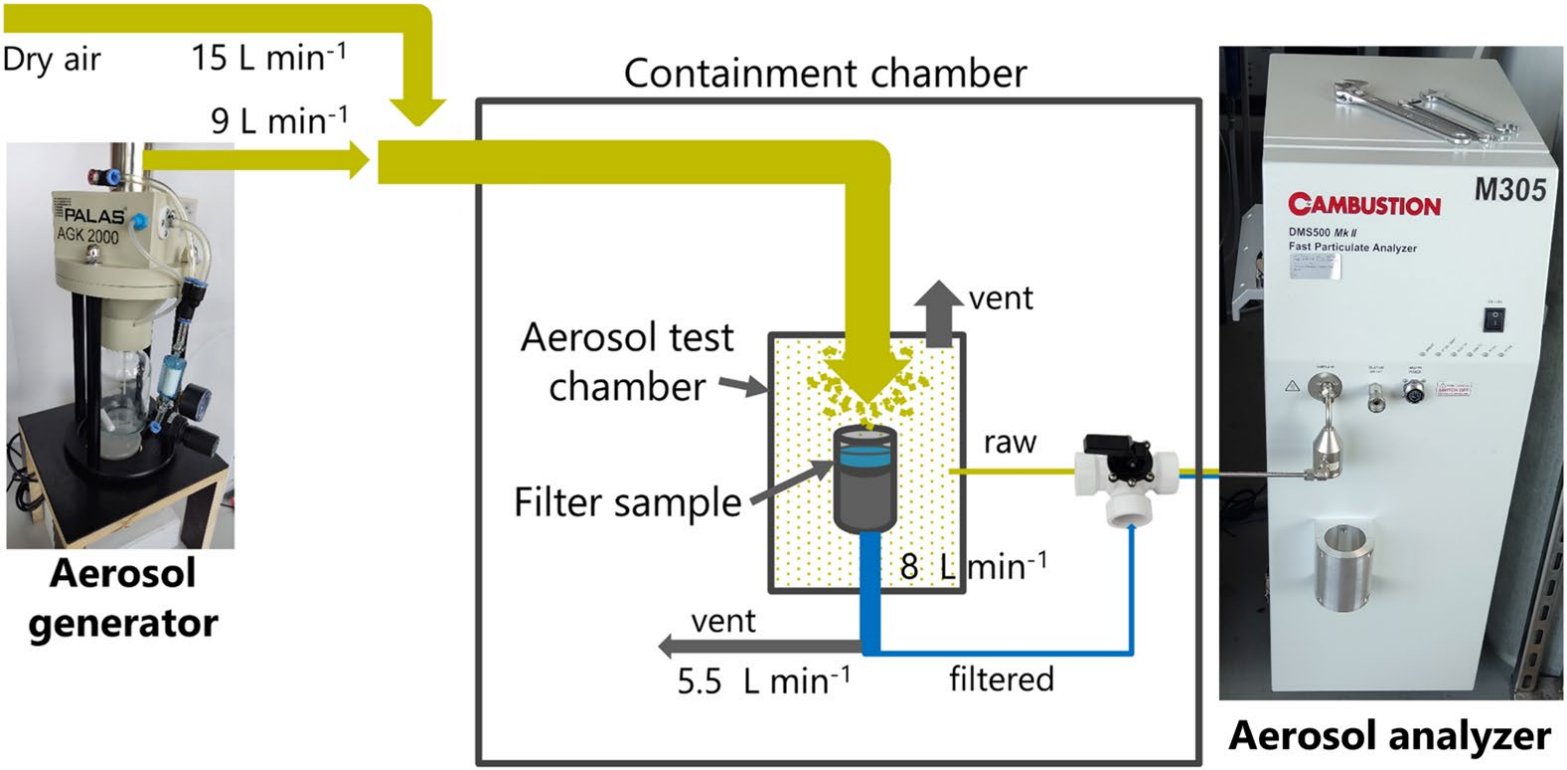
Schematic description of the filtration efficiency bench. The virus or NaCl solution was placed in the aerosol generator. A constant airflow containing viral or salt particles was generated through the specimen based on DIN 14683. The particle size distribution was measured in the particle analyzer.

To assess the fate and localization of InViS and salt particles in facemasks, the filtration efficiency set-up presented in Fig. 8 was used. By use of a pump system, a constant air flow of 8 L min^−1^ was generated through the specimen (based on EN13274-7), mimicking the human breathing volume at light physical exertion while maintaining a relative humidity in the final aerosol of 30–40% at room temperature. A circular piece of a facemask was mounted into a sample holder inside a small containment chamber and the particles diffusing through the specimen were quantified in real–time by using a particle analyzer (Cambustion DMS500). A solution of InViS in ultrapure water (10^11^ particles mL^−1^) or NaCl [2 g mL^−1^, aereosol concentration between 4 and 12 mgm^−3^ and median particle size between 60 and 100 nm] was fed to the aerosol generator (AGK 2000 Palas). As the virus stock solution was composed of virus in PBS, the aerosol solution contained 1% v/v of PBS. Therefore, not only InViS, but also PBS residues were present in the aerosol.

### Localization in facemask layers

After filtration efficiency experiments, the mask layers were peeled apart and analyzed separately to localize the InViS particles. First, scanning electron microscopy (SEM) images of the inner and outer layers provided a qualitative analysis of the particle presence on the mask fibers. For this, a Hitachi S-4800 (Hitachi High-Technologies, Canada) SEM was used. Prior to imaging, the mask layers were mounted onto SEM stubs with a conductive double sided carbon tape and sputter coated with 7 nm of gold/palladium (LEICA EM ACE600) to reduce electron charging effects. The settings for SEM imaging were an accelerating voltage of 2 kV and current flow of 10 µA. Secondly, the amount of virus on each layer was evaluated by fluorescence measurements. For this, the different mask layers were placed in 8 mL of 70% ethanol for a period of 2 h. Afterwards, 2 mL of the solution were pipetted to transparent cuvettes and fluorescence measurements were conducted with a Horiba FluoroMax SpectraFluorometer (excitation wavelength 560 nm, emission spectra between 580 and 650 nm).

### Statistical analysis

R was used for figures and statistical calculations. The statistical differences were assessed using the Student's t-test and the following symbols represent the corresponding p values: *p < 0.05, ^$^p < 0.01, ^£^p < 0.001 and ^#^p < 0.0001.

## Data Availability

Data supporting this study are provided upon reasonable request to the corresponding authors.
